# Acute Haemostatic Depletion and Failure in Patients with Traumatic Brain Injury (TBI): Pathophysiological and Clinical Considerations

**DOI:** 10.3390/jcm12082809

**Published:** 2023-04-11

**Authors:** Fabian Kockelmann, Marc Maegele

**Affiliations:** 1 Department of Surgery, Klinikum Dortmund, University Hospital of the University Witten/Herdecke, Beurhausstr. 40, D-44137 Dortmund, Germany; 2 Institute for Research in Operative Medicine (IFOM), University Witten/Herdecke, Campus Cologne-Merheim, Ostmerheimerstr. 200, D-51109 Köln, Germany; 3 Department of Trauma and Orthopedic Surgery, Cologne-Merheim Medical Center (CMMC), University Witten/Herdecke, Campus Cologne-Merheim, Ostmerheimerstr. 200, D-51109 Köln, Germany

**Keywords:** coagulopathy, traumatic brain injury, diagnostics, mechanisms, treatment

## Abstract

Background: Because of the aging population, the number of low falls in elderly people with pre-existing anticoagulation is rising, often leading to traumatic brain injury (TBI) with a social and economic burden. Hemostatic disorders and disbalances seem to play a pivotal role in bleeding progression. Interrelationships between anticoagulatoric medication, coagulopathy, and bleeding progression seem to be a promising aim of therapy. Methods: We conducted a selective search of the literature in databases like Medline (Pubmed), Cochrane Library and current European treatment recommendations using relevant terms or their combination. Results: Patients with isolated TBI are at risk for developing coagulopathy in the clinical course. Pre-injury intake of anticoagulants is leading to a significant increase in coagulopathy, so every third patient with TBI in this population suffers from coagulopathy, leading to hemorrhagic progression and delayed traumatic intracranial hemorrhage. In an assessment of coagulopathy, viscoelastic tests such as TEG or ROTEM seem to be more beneficial than conventional coagulation assays alone, especially because of their timely and more specific gain of information about coagulopathy. Furthermore, results of point-of-care diagnostic make rapid “goal-directed therapy” possible with promising results in subgroups of patients with TBI. Conclusions: The use of innovative technologies such as viscoelastic tests in the assessment of hemostatic disorders and implementation of treatment algorithms seem to be beneficial in patients with TBI, but further studies are needed to evaluate their impact on secondary brain injury and mortality.

## 1. Introduction

**Figure 1:**
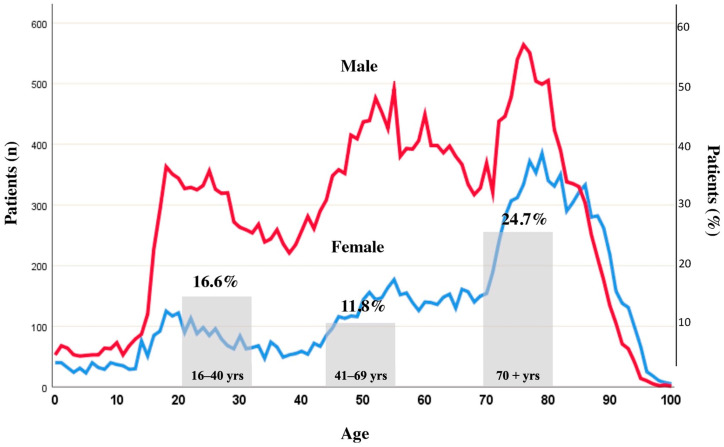
Trimodal age distribution of moderate to severe traumatic brain injury in men and women: 1. first peak: marked difference in incidence between the sexes, starting at puberty and rising until age 18 (driver’s license); “testosterone effect” > “young risk-takers”; male > female; 2. second peak: mid-fifties (“older risk-takers”; work accidents); 3. third peak: late seventies (incidence in the two sexes is now more nearly equal; mainly falls) and frequency of coagulopathy upon admission for three age groups according to the Berlin definition of coagulopathy, e.g., PTT ≥ 40 s and/or INR ≥ 1.4. First published by Maegele et al. [6], with permission from Deutscher Ärzteverlag GmbH; Reprinted from Maegele et al. [7], adapted with permission from Wolters Kluwer Health, Inc., Alphen aan den Rijn, The Netherlands.

Traumatic brain injury (TBI) remains a significant health problem of our times with high socioeconomic burden [1,2]. The primary injury is usually due to the physical impact on the brain itself with contusional injury and hemorrhagic lesions through cerebral blood vessel disruptions and/or blood-brain barrier (BBB) breakdown [3]. Further clinical courses may be deteriorated by either pre-existing or developing alterations in the sensitive balance between bleeding and thrombosis, resulting in overall impaired hemostasis, in the following referred to as coagulopathy, with significant deterioration of injuries sustained [3]. Over the recent years, TBI epidemiology has seen a shift towards older ages with low falls as the most common cause of injury in high-income countries and an increasing frequency of contusional injuries [4] along with traumatic intracranial hemorrhage (ICH), including progression in more severe cases [5]. Figure 1 displays the current trimodal distribution of TBI in relation to age and the coincidence of haemostatic failure [6].

## 2. Frequency of Haemostatic Disorders after Isolated TBI upon Admission and the Role of Preinjury Anticoagulant and/or Antiplatelet Therapies

Recent observational data from the pan-European CENTER-TBI study on patients with isolated TBI have confirmed laboratory coagulopathy in 16 percent of patients with no intake of antiplatelet drugs and/or anticoagulants before trauma versus 34 percent of patients with pre-injury antiplatelet and/or anticoagulant therapy upon hospital admission [8]. In general, there is an elevated incidence of coagulopathy in patients with increased severity of the injury and penetrating versus blunt trauma [9]. Preinjury intake of warfarin was linked to a two-fold increase in poor outcomes [10], while preinjury intake of antiplatelet drugs was associated with increased traumatic intracranial hemorrhage (ICH) to the same magnitude [11]. A recent propensity-matched cohort analysis, also from the CENTER-TBI study, has confirmed the relation of preinjury antithrombotic agents with greater expansion of extra-axial lesions, increased rates of hemorrhagic progression, and higher risk of delayed traumatic ICH [12]. Novel oral anticoagulants (NOACs) are currently superseding vitamin K antagonists (VKA) in the prevention of thromboembolic events, but their role in traumatic ICH still needs to be defined. In theory, these agents may be more secure in the brain because of the tissue factor-rich environment and retained function of factor VII, but this remains to be determined. To date, NOAC-related bleedings in trauma patients have been investigated in two prospective trials, but different results have been reported [13,14]. A mono-centric retrospective trial has assessed hematoma expansion in patients with traumatic ICH on NOACs versus vitamin K antagonists and reported a higher rate for NOACs. The overall hematoma expansion rate in this study across all patients was 36% [15]. Table 1 shows the prevalence of coagulopathy in isolated TBI.

## 3. The Clinical Pattern of Coagulopathy after TBI

**Figure 2:**
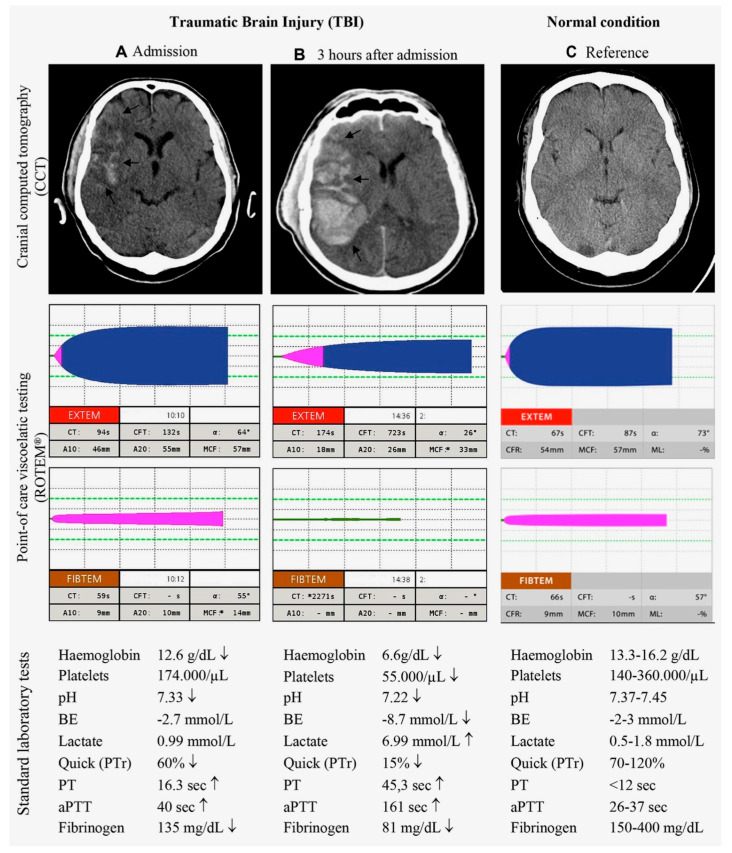
Clinical example of a patient with severe TBI and initial coagulopathy who developed complicating subsequent hypotensive (multifactorial) coagulopathy with deteriorating ICH within three hours of hospital admission. Shown are cranial computed tomographies of the brain (CCTs), results from viscoelastic (ROTEM^®^), and standard laboratory and conventional coagulation assays (CCAs) upon admission (**A**) and after three hours of admission (**B**). A normal reference condition is displayed for comparison (**C**). Viscoelastic assays after three hours of admission indicate delayed and insufficient clotting, as reflected by prolonged initiation times and reduced clot amplitudes. The flat line in the ROTEM^®^ FIBTEM channel reflects the complete absence of fibrin polymerization. Results from standard laboratory assays and CCAs three hours after admission display signs of shock with deranged coagulation along with hypofibrinogenemia and thrombocytopenia. aPTT = activated Partial Thromboplastin Time; BE = base excess; PT = Prothrombin Time; PTr = Prothrombin ratio. First published by Maegele et al. [3], with permission from Elsevier; Reprinted from Maegele et al. [7], with permission from Wolters Kluwer Health, Inc.

**Figure 3:**
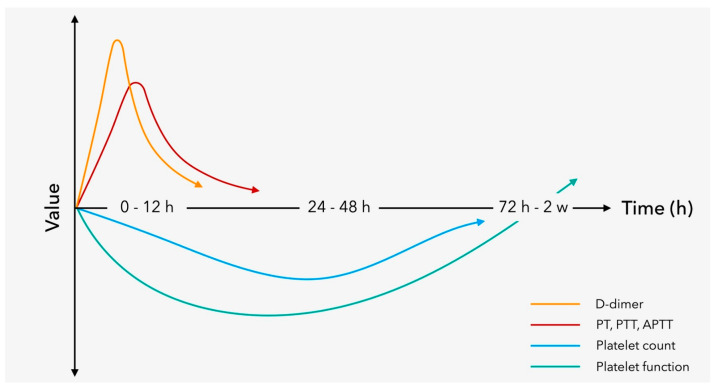
Conceptual timeline of hemostatic disruptions after TBI based upon changes in conventional coagulation assays (CCAs). Reprinted from Fletcher-Sandersjöö et al. [37], under CC BY-4.0, http://creativecommons.org/licenses/by/4.0/ (assessed on 3 January 2023).

In the first 24 h after admission to the hospital, we can observe a doubling in the prevalence of coagulopathy in patients with TBI [23], and a comparable number of patients may develop injury progression like hemorrhage or ICH with coagulopathy within 6 to 48 h [34]. Taken together [23,34], within the first 72 h after trauma, patients are at risk for developing coagulopathy after TBI [35]. A clinical example of a patient with isolated TBI and rapid evolvement of coagulopathy, including dramatic hemorrhagic progression within three hours of hospital admission, is shown in Figure 2. Retrospective evidence suggests an inverse relationship between the onset of coagulopathy and injury severity with persisting disruptions of the coagulation system until day three after injury or even longer [9,32]. In contrast, lesion progression in mild TBI usually stops within 24 h of injury [36]. While the pathophysiology behind these disruptions remains largely unexplained, there may be a coincidence of both hypo- and hypercoagulable states, with a predominance of the first during the acute phase and the latter during later stages [3,5]. A conceptual framework of the laboratory changes based upon conventional coagulation assays to reflect early coagulopathy after TBI for the first 72 h after injury is depicted in Figure 3 [37].

## 4. Coagulopathy as a Powerful Predictor for Prognosis

The occurrence of coagulopathy upon hospital admission has frequently been identified as a powerful predictor for prognosis after TBI [5]. Aggregated data from a series of clinical studies suggest increased mortality up to nine-fold and risk of poor outcomes up to thirty-fold [16]. Hemostatic disruptions based upon conventional coagulation assays were associated with progressive hemorrhagic injury (PHI) and ICH [38,39], while impaired clot formation observed in functional clotting assays was linked to a five times higher mortality from ICH progression [34]. Several independent risk factors for acute coagulopathy in isolated TBI could be identified, which include systolic hypotension, base excess, hypothermia, reduced Glasgow Coma Scale (GCS) on presentation and hypoxia [8]. Low thrombocyte counts and/or impaired platelet function can further contribute to bleeding, but this is still a work in progress [29].

## 5. Potential Mechanisms of Hemorrhagic Progression and Coagulopathy after TBI

### 5.1. Hemorrhagic Progressions and New Lesions

In general, coagulopathy does not cause hemorrhage in the absenteeism of any (micro)vascular injury or failure, including blood-brain barrier (BBB) malfunction or breakdown. Experimental imaging studies have shown secondary bleeding from ruptured arterioles and venules localized to the periphery of existing hematomas and into congested areas of perihematoma tissues [40,41]. Alterations in histopathological blood clot morphology due to active or ongoing bleeding after ICH have also been associated with early hematoma growth [42]. It has been concluded from these findings that early hemorrhagic progression results primarily from bleeding into acutely formed congested tissue layers around the initial hematoma [43]. Factors that may contribute to these developments include local tissue pressure, local fibrinolysis, plasma protease induction and activation of secondary inflammatory mechanisms [44]. Noteworthy, hemorrhagic progression is not only limited to the growth of primary contusions but may also occur as delayed, non-contiguous hemorrhagic lesions at distant locations [45,46]. The latter has been linked to the activation of remote vessel-bound mechanosensitive receptors that trigger further downstream mechanisms to induce capillary fragmentation [45].

### 5.2. Coagulopathy after TBI

**Figure 4:**
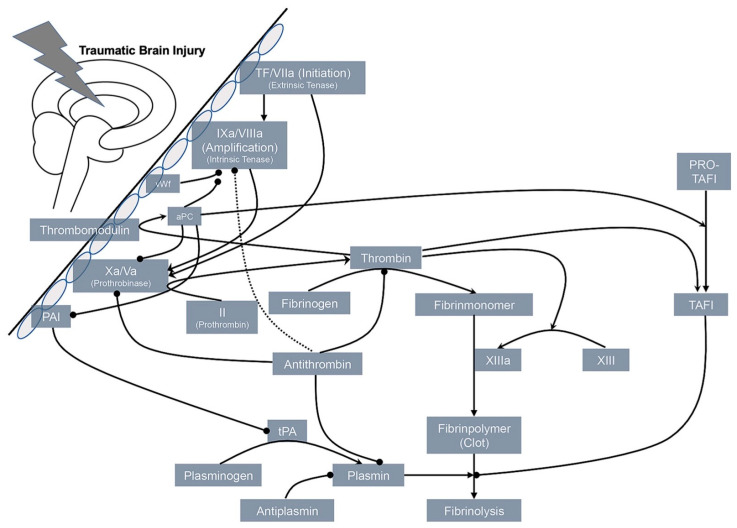
Summary of potential interactions and pathways involved in the development of hemostatic failure and coagulopathy after TBI. The exact nature of hemostatic disruptions observed after TBI remains elusive, but current evidence suggests the presence of both a hyper- and hypocoagulable state with possible overlap and lack of distinction between phases and states. Arrows indicate activation; bold circles inhibition. Reprinted from Maegele et al. [7], with permission from Wolters Kluwer Health, Inc.

Direct injury to the blood vessel or defragmentation from microvascular failure triggers the release of tissue factor (TF) which promotes the extrinsic coagulation pathway [47] with consumption, disseminated intravascular coagulation (DIC) and further bleeding [48,49]. Further molecules were postulated to play a significant role in local and systemic coagulopathy in TBI, for example, tissue plasminogen activator (tPA) and urokinase plasminogen activator (uPA) [50]. Patients who developed DIC early after isolated TBI showed characteristic features, such as low platelet counts, signs of consumption coagulopathy and insufficient coagulation control by antithrombin [48]. The observation of high levels of fibrin degradation products (FDP) and D-dimer, together with increased FDP/D-dimer ratios, indicate that DIC may be considered a fibrinolytic phenotype. In addition, D-dimer levels reach their maximum three hours after trauma, when bleeding tendency is maximum as well [51]. The occurrence of DIC coincidences with clinical features of systemic inflammatory response syndrome (SIRS), single- and multiorgan dysfunction and need for blood transfusion, in particular in the presence of hyperfibrinolysis, with overall worse outcomes. Tissue factor (TF) is available in its blood-borne soluble form and its free form, which is located on the surface of activated platelets and through microparticles. Both forms could promote continuous amplification. A recent study could confirm early enhanced activation, insufficient inhibition, exacerbation of thrombin generation and following fibrinolysis in patients with isolated TBI [33]. Thrombin has been identified as a strong inducer for the release of microparticles from various origins, such as thrombocyte and endothelial cells, thereby enlarging procoagulant surfaces and providing a pool for circulating TF [52]. Through its receptor PAR-1 on endothelial cells, thrombin may also contribute to vasoconstriction, which limits the bleeding process but can also worsen ischemia in TBI [53]. Figure 4 displays some potential interactions and pathways leading to coagulopathy after TBI.

### 5.3. Traumatic Brain Injury and Shock

Hypoperfusion and shock play a pivotal role in the development of hemostatic failure [8,31]. Shock and hypoperfusion trigger the activation of coagulation and complement systems with (i.) endothelial shedding, (ii.) activation of the protein C pathway with hyperfibrinolysis and the inhibition of coagulation factors FVa and FVIIIa, and (iii.) inflammation. Ischemia, as well as hypoperfusion, further aggravate coagulopathy and TBI in general through secondary mechanisms, which are known as vasoconstriction, endothelial dysfunction and swelling, systemic hypotension and microvascular occlusion by thrombocyte or leukocyte activation, adhesion or aggregation [38,54]. Contusional brain injuries may also liberate tissue-type (tPA) and urokinase-type plasminogen activators (uPA), thereby promoting local fibrinolysis [55]. The delicate “cross-talk” between the different systems activated during TBI, e.g., the sympathetic nervous system (SNS), coagulation, inflammation and complement, including time- and context-dependent amplification and inhibition, needs to be further investigated [56].

### 5.4. The Role of Platelets

The role of platelets in the context of TBI-associated coagulopathy remains poorly defined. It is understood that low platelet counts and platelet dysfunction contribute to hemostatic failure and disease progression [57,58]. While thrombocyte dysfunction, mostly associated with significant inhibition for arachidonic acid (AA) and adenosine diphosphate (ADP), has been observed with platelet counts within reference ranges or in the absence of antiplatelet medication [58], low platelet counts together with spontaneous platelet aggregation in non-bleeding TBI patients may also indicate at least partial or temporal hyperactivity. Experimental studies have demonstrated intravascular microthrombosis, resulting in reduced pericontusional blood flow. One explanation for this scenario is that brain-derived platelet-activating factor (PAF) promotes hypoxia-associated BBB breakdown. This process results in other brain-derived liberated pro-coagulative substances and additional PAF. The role of important platelet ligands, for example, Willebrand factor (vWF), which capture an additional number of thrombocytes to the perturbated endothelium or to the exposed subendothelial matrix resulting in local in-situ thrombosis and thromboembolism in the downstream microvasculature, still needs to be further investigated.

### 5.5. Experimental Models

In vivo and in vitro models are very useful in TBI research. Common in vivo models are weight drop, fluid percussion, controlled cortical impact injury, blast injury, penetrating ballistic-like brain injury and acceleration. In contrast to these, there are three usually used in vitro models called immortalized cell lines, dissociated primary cultures and organotypic cultures from different individuals [59]. The most widely used in vivo model is the fluid percussion model. The target animal, for example, mice, needs a craniectomy before the fluid pressure device applies an exact pressure pulse on the intact dura by using a system of fluid that transmits the mechanical energy [60]. There are two other approaches to applying controlled energy to the dura: In controlled cortical impact injury, energy is applied directly by a machine to the skull [61], whereas, in the weight drop model, a rod with a specific weight is dropped on the animal’s skull [62].

In contrast to in vivo models, in vitro models have better reproducibility but transfer from cell culture to clinical reality is difficult because of the complexity of TBI. Immortalized cell lines are useful for examining cellular proliferation, differentiation and molecular pathways in cells, and there are established cell lines for specific research fields [63]. Dissociated primary cultures are useful for gaining insights into the morphology and metabolism of specific cells in different environments after the cells have been isolated from a living organism and were cultivated for some time [59,64]. A disadvantage of this technique is the investigation of cells outside their formation. This fact is considered in organotypic models: A slice of brain tissue is cultivated, and different types of drugs or cell grafts can be added either in the growth medium or in the tissue slice with a needle [65]. The advantage of this method is that the in vivo environment of cells is received [65].

## 6. Novel Diagnostic Approaches to Coagulopathy

**Figure 5:**
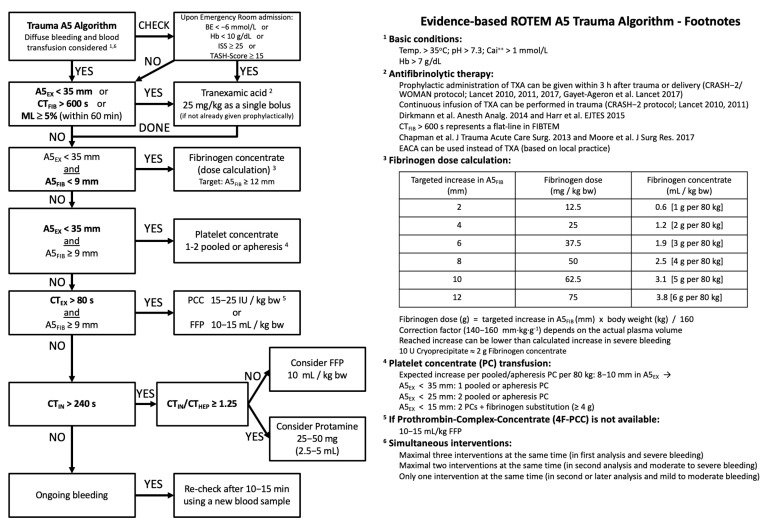
Exemplar algorithm to guide hemostatic therapies by the use of functional viscoelastic testing results (ROTEM^®^) successfully implemented at centers formerly naïve to this technology. Test results were available for clinical decision-making within a median of 15 min of blood sampling. When the algorithm recommended specific interventions and was followed, thromboelastometric test results improved significantly by the second blood sampling performed 30–60 min after decision-making and at least 10 min after the intervention (adapted from [66]). A10 = Clot amplitude after 5 and 10 min (in mm); CT = Clotting time (in seconds); Ex = ROTEM^®^ EXTEM assay; FFP = Fresh Frozen Plasma; Fib = ROTEM^®^ FIBTEM assay; g = gram; IU = International Units; ML = Maximum lysis (in percent); PCC = Prothrombin Complex Concentrate.

Functional hemostatic assays, for example, viscoelastic tests (ROTEM^®^ and TEG^®^) with platelet function assays and thrombin generation tests, may have advantages over conventional coagulation assays (CCAs) in the early detection of hemostatic abnormalities after TBI and in monitoring and guiding subsequent therapies [3,25,66,67]. In thromboelastography (TEG^®^) or thromboelastometry (ROTEM^®^), shear forces are measured optically or electromechanically between a wire and a cup in whole blood samples when pro-coagulatory agents are added. Depending on the clotting process, information about platelet function, fibrin polymerization and fibrinolysis is provided within minutes. While CCAs only reflect the initiation process of coagulation and only a small part of thrombin generation, “point of care” viscoelastic assays provide timely and more detailed information on the overall hemostatic potential [25,66]. They characterize coagulopathy patterns in TBI patients, and abnormal profiles have been associated with poor outcomes [68]. In addition, specific viscoelastic subtests (ClotPro^®^ Ecarin clotting time and Russel Viper venom/RVV clotting time) are sensitive to the detection of clinically-relevant NOAC plasma drug levels [69]. Viscoelastic assays can be used bedside, in the resuscitation room, the operation theatre and/or the intensive care unit, and results with readout after five minutes of test begin currently form the backbone of many clinical algorithms for “goal”-directed hemostatic therapies after TBI [3,66]. A clinical example of such an algorithm for the specific application in TBI patients is shown in Figure 5. A recent feasibility study in clinical centers initially naïve to the technology confirmed rapid adoption and adherence after short-term training to the technology and to a corresponding treatment algorithm [66]. “Point-of-care” platelet function tests can be helpful in detecting platelet dysfunction or therapeutic platelet inhibition. This seems to be useful because ADP inhibition has been shown to correlate with injury severity in TBI [70]. However, these tests require sufficient platelet numbers, and their routine availability outside the research setting is limited.

## 7. Treatment Approaches to Coagulopathy after TBI

### 7.1. Reversal of Iatrogenic Anticoagulation

Rapid therapy of hemostatic abnormalities in the acute phase after TBI has been independently associated with survival in TBI [71]. For the emergency reversal of preinjury medication with vitamin K-associated anticoagulants, e.g., warfarin, the early administration of prothrombin complex concentrate (PCC) is beneficial and recommended by current guidelines [72,73]. For the emergency reversal of NOACs in case of life-threatening bleeding, two specific antidotes have been approved: (i.) idarucizumab for the reversal of dabigatran, and (ii.) andexanet alfa for the reversal of apixaban and rivaroxaban [74]. In case of non-availability, nonspecific prohemostatic agents, including (activated) prothrombin complex concentrate (PCC) and tranexamic acid (TXA), have been used off-label for NOAC reversal and thrombin generation. Table 2 gives advice for the management of acute reversal of preinjury anticoagulatoric medication in adults with intracranial hemorrhage (ICH) along with TBI.

Whether the administration of platelet concentrates in TBI and moderate thrombocytopenia will be beneficial is still not clarified. The administration of platelet concentrates in TBI and moderate thrombocytopenia remains elusive. The use of platelet concentrates has not resulted in better outcomes to date [75]. In the subgroup of patients with spontaneous ICH under antiplatelet drugs, standard care was better [76]. A recent meta-analysis (although based on only small and observational studies) failed to show a mortality benefit in traumatic ICH while on antiplatelet therapy [77]. The administration of platelet concentrates in TBI patients on antiplatelet therapy appears plausible, but current evidence is still low, and possible side effects must be considered.

### 7.2. Conventional Blood Products

In everyday clinical practice, packed red blood cell concentrates (pRBCs) are the most transfused blood products, although evidence for transfusion threshold in patients with TBI is still missing [78]. Several randomized trials have been conducted, but neither maintaining hemoglobin levels > 10 g/dL nor the administration of erythropoietin could be linked to improved neurological outcomes at six months. A liberal transfusion regime with a target hemoglobin level of 10 g/dL versus 7 g/dL was associated with complications like hemorrhagic progression [79,80]. At present, red blood cell transfusions should be avoided as far as possible, and the transfusion threshold should be set low unless there is evidence of poor anemia tolerance [78].

Experimental resuscitation with fresh frozen plasma concentrates (FFP) in different models of TBI and hemorrhagic shock was associated with reduced lesion size, downregulation of inflammatory genes, and improved protein expression profiles for blood–brain barrier (BBB) integrity [81,82]. The latter could even be improved through the addition of valproic acid, most likely secondary to the activation of key pathways for endothelial function [82,83]. In this context, retrospective clinical data was able to confirm the beneficial effects of early FFP administration according to better in-hospital survival when FFPs are given within four hours of injury, and multifocal intracranial hemorrhage is observed [84]. Results from an exploratory sub-study of the prospective randomized PAMPer trial revealed an improved 30-day survival in patients with TBI and a prehospital Glasgow Coma Scale (GCS) score < 8 with two units of prehospital plasma even after adjustment for multiple confounders and injury severity [85]. In patients with severe TBI [86] or TBI in combination with moderate coagulopathy, transfusion of FFPs alone or in addition to pRBCs seems not to be beneficial because of adverse events and poorer functional outcomes [75].

### 7.3. Tranexamic Acid (TXA)

The pragmatic randomized controlled Clinical Randomisation of an Antifibrinolytic in Significant Haemorrhage (CRASH)-3 trial demonstrated, for the first time, an improvement in a pharmacological intervention for the least-specific TBI sub-groups [87]. A statistically significant reduction in the risk of head injury-related mortality was reported in patients with mild and moderate TBI when TXA was administered within three hours of TBI. The CRASH-2 IBS (Intracranial Bleeding Study) was a nested trial into CRASH-2, and the aim of the study was to determine the effect of a short course of TXA on intracranial hemorrhage and focal cerebral ischemia [88]. The focus of the study was to investigate the increase in the size of intracranial hemorrhage on CT scans between admission and follow-up at 24–48 h. The analysis of 123 patients suggested that admission of TXA was associated with fewer deaths, decreased hemorrhage growth and fewer focal ischaemic lesions, but with substantial uncertainties remaining. Risks and benefits of early TXA administration, even before hospital admission, were investigated in a prospective phase II trial in patients with expected moderate to severe TBI without shock but did not improve six-month neurologic outcomes (GOSE-E) as a primary outcome measure [89]. Interestingly, in patients in whom intracranial hemorrhage (ICH) was detected in the hospital, the 28-day mortality was 18% in those patients who had received TXA at 2 g bolus out-of-hospital, versus 26% in those who had received the classical CRASH-2 dosing regimen, versus 27% in the placebo group. Retrospective analysis of observational data from patients with only severe TBI showed increased mortality with 1 g TXA prehospital after controlling for confounders [90]. Taken together, the effect of TXA appears to differ in subgroups and depends on both the dose and severity of bleeding. In any case, the potential and dose-dependent risk for thromboembolic events with TXA needs to be considered.

### 7.4. Goal-Directed Therapies

Viscoelastic assays (ROTEM^®^ and TEG^®^) have successfully been integrated into diagnostic and therapeutic approaches in high-risk patients with active hemorrhage and furthermore in patients with TBI [25,66,72,91]. Meanwhile, an increasing number of clinical guidelines support their use with considerable level of recommendation but this is against still existing low-quality evidence in this population [68,72,91,92]. Implementation of such algorithms, even in centers naïve to this approach, is timely and feasible with good adherence, reduced turnaround times for test results and accelerated clinical decision-making [66]. If specific recommendations provided by the algorithm were followed, viscoelastic test results improved significantly by the time when the second blood sample was drawn. A prespecified subgroup analysis of the recently published pragmatic iTACTIC-trial demonstrated that the use of viscoelastic tests and treatment according to specific trauma hemorrhage protocols were beneficial in two of three patients with severe TBI related to reduced mortality and massive transfusion at 24 h [93]. In the same study, 28-day mortality was significantly reduced with the viscoelastic-based intervention versus the conventional coagulation assay-guided intervention for acute hemostatic control (44% versus 74% mortality). Severe TBI patients in the treatment group guided by viscoelastic testing received more often fibrinogen and/or platelets in contrast to patients in the CCA-guided treatment group, an observation that may explain the difference in survival between the two study arms. In this trial, the potential for viscoelastic-guided interventions to improve mortality appeared to increase with injury severity [93]. Another subgroup analysis of severe TBI patients from another pragmatic randomized trial using a viscoelastic resuscitation strategy, but yielding only very small patient numbers, did not report reduced mortality [94]. This TBI subgroup was not specified a priori, so only nine TBI patients were included in the viscoelastic-based treatment arm resulting in too little statistical power for mortality. When data for severe TBI patients from both trials were aggregated into a meta-analysis, a significant 28-day mortality benefit in favor of the viscoelastic-guided approach was seen, but with a “very low” study quality assessment. In a prospective single-center and case-control study involving 134 patients with isolated TBI and need for craniotomy, viscoelastic testing was associated with more consistent coagulation management along with improved clot quality, reduced hemorrhagic progression and less neurosurgical reinterventions [95].

## 8. Limitations

This review is intended for clinical practitioners and provides an overview of current knowledge of pathophysiology and therapeutic strategies in TBI, given therapeutic strategies are based on data or clinical experience of their authors. Nevertheless, treatment options mentioned in this review need further research, and big cohort studies are missing, especially for new reversal drugs. Potential side effects from thromboembolic events to death have been reported. In everyday clinical practice, this review can give some advice for therapeutic considerations, but individual risk-benefit analyses are needed for every patient. This review does not relate to patients with pre-existing hemostatic disorders or children suffering from TBI.

## 9. Conclusions

The use of viscoelastic tests, in addition to conventional coagulation laboratory parameters, seems to be beneficial in patients with TBI and especially in patients with anticoagulatoric medication. Because of the aging population, more older patients with anticoagulatoric medication will suffer from TBI. Diagnostic and therapeutic approaches will be needed to improve survival and functional outcome. Point-of-Care diagnostic and Goal-directed-therapies seem to be promising fields of research. Further studies are needed to evaluate their impact on secondary brain injury and mortality and to define precise therapeutic thresholds.

## Figures and Tables

**Table 1:** Prevalences of coagulopathy in isolated traumatic brain injury (iTBI), including outcome in the presence of coagulopathy.

Study	No. of Patients	Definition of TBI	Definition of Coagulopathy	Prevalence of Coagulopathy (%)	Mortality with Coagulopathy (%)
Harhangi [16] *	5357	Heterogeneous	Heterogeneous	32.7 (10–97.5)	51 (25–93)
Epstein [17] **	7037	Heterogeneous	Heterogeneous	35.2 (7–86.1)	17–86
Zehtabchi [18]	224	AIS_head_ > 2 and/or any intracranial hematoma on CT	INR > 1.3 or PTT > 34 s	17 (8–30)	-
Talving [19]	387	AIS_head_ ≥ 3 and extracranial AIS < 3	Platelets < 100,000 mm^3^ or INR > 1.1 or aPTT > 36 s	34	34.7
Lustenberger [9]	278	AIS_head_ ≥ 3 and extracranial AIS < 3	Platelets < 100,000 mm^3^ and/or INR > 1.4 and/oraPTT > 36 s	45.7	40.9
Lustenberger [20]	132	AIS_head_ ≥ 3 and extracranial AIS < 3	Platelets < 100,000 mm^3^ or INR > 1.2 or aPTT > 36 s	36.4	32.5
Wafaisade [21]	3114	AIS_head_ ≥ 3 and extracranial AIS < 3	Quick (PTR) < 70% and/or platelets < 100,000/mL	22.7	50.4
Chhabra [22]	100	GCS < 13	Fibrinogen < 200 mg/dL	7	-
Greuters [23]	107	Brain tissue injury on CT and extracranial AIS < 3	aPTT > 40 s and/or INR > 1.2 and/or platelets < 120 × 10^9^/L	24 (54 ^#^)	41
Shehata [24]	101	iTBI on admission brain CT	INR ≥ 1.2, PT > 13 s, d-dimer positive, platelets < 100 × 10^3^/CC	63	36
Schöchl [25]	88	AIS_head_ ≥ 3 and extracranial AIS < 3	Quick (PTR) < 70% and/or aPTT > 35 s and/or fibrinogen < 150 mg/Dl and/or platelets < 100 × 10^9^/L	15.8	50
Franschman [26]	226	iTBI on CT and extracranial AIS <3	aPTT > 40 s and/or PT > 1.2 and/or platelets < 120 × 10^9^/L	25 (44 ^#^)	33
Genet [27]	23	AIS_head_ ≥ 3 and extracranial AIS < 3	aPTT > 35 sand/orINR > 1.2	13	22
Alexiou [28]	149	iTBI with exclusion of multisystem trauma	aPTT > 40 s and/or INR > 1.2 and/or platelets < 120 × 10^9^/L	14.8 (22.8 ^#^)	-
Joseph [29]	591	AIS_head_ ≥ 3 and extracranial AIS < 3	INR ≥ 1.5 and/or PTT ≥ 35 s and/or platelets ≤ 100 × 10^3^/mL	13.3	23
Epstein [17]	1718	AIS_head_ ≥ 3 and extracranial AIS < 3	INR ≥ 1.3	7.7	45.1
De Oliveira Manoel [30]	48	AIS_head_ ≥ 3 and extracranial AIS < 3	INR ≥ 1.5 and/or aPTT ≥ 60 s and/or platelets < 100 × 10^3^/mm^3 §^	12.5	66
Dekker [31]	52	AIS_head_ ≥ 3	INR ≥ 1.2 and/or aPTT ≥ 40 s and/or platelets < 120 × 10^9^/L	42	45.5
Yuan [32]	2319	Intracranial injury on CT and extracranial AIS < 3	INR > 1.25 and/or PT > 14 s and/or aPTT > 36 s and/or platelets < 100 × 10^9^/L	18.6	17.6
Albert [33]	561	iTBI on admission brain CT	INR ≥ 1.27 and/or PT ≥ 16.7 s and/or aPTT > 28.8 s	41.6%	61.1%
Böhm [8]	598	iTBI on CT and no extracranial injuries	INR > 1.2 and/or aPTT > 35 s and/or fibrinogen < 150 mg/dL and/or platelets < 100 × 10^3^/nL	19.6	-

AIS = Abbreviated Injury Scale; aPTT = activated partial thromboplastin time; CT = computed tomography; GCS = Glasgow Coma Scale; INR = International Normalized Ratio; PT = prothrombin time (PTR = prothrombin ratio/quick); TBI = traumatic brain injury; * Meta-analysis (1966–2007) with *n* = 34 studies included; ** Meta-analysis (1990–2013) with *n* = 22 studies included; # after 24 h; § additional abnormal coagulation tests: fibrinogen ≤ 1.0 g/L, any clotting factor < 0.5 (<50% activity) and abnormal viscoelastic test results. First published by Maegele et al. [3], with permission from Elsevier; Reprinted from Maegele et al. [7], with permission from Wolters Kluwer Health, Inc.

**Table 2:** Current options for the reversal of antithrombotic agents in adult patients with TBI and traumatic intracranial hemorrhage (ICH).

Antithrombotic	Strong Recommendation with Moderate-to-High Quality Evidence	Conditional Recommendation with Low-to-Moderate Quality Evidence
Vitamin K antagonists (VKAs)	Vitamin K for INR reversal in VKA-associated ICH as soon as possible or with other reversal agents 3- and 4-factor PCC iv be preferred to FFP in VKA-associated ICH and INR ≥ 1.4; dosing on weight, INR and type of PCC. Monitoring via repeated INR after PCC administration (15–60 min) and every 6–8 h for 24–48 h; subsequent treatment according to follow-up INR as repeated dosing may increase thrombotic and DIC risk (Good practice statement) Treatment with vitamin K and FFP is recommended over no treatment!	If INR ≥ 1.4 vitamin K 10 mg iv with subsequent treatment according to follow-up INR; if repeated INR ≥ 1.4 within 24–48 h redosing with vitamin K 10 mg iv (Good practice statement) or if treated with PCC and repeated INR ≥ 1.4 within first 24–48 h further correction with FFP Fresh frozen plasma/FFP (10–15 mL/kg iv) along with one dose of vitamin K 10 mg iv if PCC is not available/contraindicated
Direct factor Xa inhibitors	Andexanet alfa (400–800 mg as an initial bolus followed by 4–8 mg/min over 120 min (480–960 mg); approved for adults treated with direct factor Xa inhibitors apixaban and rivaroxaban if rapid reversal is indicated due to life-threatening or uncontrolled bleeding!	4-factor PCC or activated PCC (4-factor PCC 50 U/kg iv or aPCC (FEIBA) 50 U/kg iv) if ICH occurred within 3–5 terminal half-lives of drug exposure or in the context of liver failure Activated charcoal (50 g) within 2 h of drug ingestion to intubated ICH patients with enteral access and/or low risk of aspiration
Direct thrombin inhibitors (DTIs)	Idarucizumab (2 × 2.5 g/50 mL) to ICH associated with dabigatran when administered within 3–5 half-lives and no renal failure and in renal failure with continued drug exposure beyond normal 3–5 half-lives	If idarucizumab is not available or in case of overdose, consider hemodialysis; consider redosing idarucizumab and repeated hemodialysis in ongoing bleeding 4-factor PCC or activated PCC (4-factor PCC 50 U/kg iv or aPCC (FEIBA) 50 U/kg iv) if idarucizumab is not available or if ICH with DTIs other than dabigatran and if DTI was administered within 3–5 half-lives and absence of renal failure or in renal failure with continued drug exposure beyond normal 3–5 half-lives Activated charcoal (50 g) within 2 h of drug ingestion to intubated ICH patients with enteral access and/or low risk of aspiration
Unfractionated heparin	Protamine sulfate iv for heparin reversal with dosing according to heparin dose over the preceding 2–3 h; protamine sulfate 1 mg for every 100 U heparin given over the preceding 2–3 h with a maximum single dose of 50 mg	If aPTT remains elevated, repeated protamine sulfate at 0.5 mg per 100 U of heparin
Low Molecular Weight Heparin (LMWHs)	Protamine sulfate slowly IV over 10 min in the following dosing, if (a.) enoxaparin was given within 8 h 1 mg protamine per 1 mg of enoxaparin administered (maximum single dose 50 mg), if (b.) enoxaparin was given within 8–12 h 0.5 mg protamine per 1 mg enoxaparin (maximum single dose 50 mg). The following dosing applies for dalteparin, nadroparin and tinzaparin: Protamine sulfate 1 mg per 100 anti Xa U of LMWH administered during the past 3–5 half-lives with maximum single dose 50 mg. Only minimal effect on reversal > 12 h from dosing!	Redosing protamine sulfate (0.5 mg per 100 anti-Xa U of LMWH or per 1 mg of enoxaparin) if life-threatening bleeding continous or in renal insufficiency Recombinant factor VIIa (rFVIIa 90 μg/kg iv) if protamine is contraindicated Reversal of danaparoid with rFVIIa in the context of ICH
Pentasaccharides	No recommendation	Activated PCC (aPCC (FEIBA) 20 U/kg iv) for pentasaccharide reversal Recombinant factor VIIa (rFVIIa 90 μg/kg iv) if aPCC is contraindicated/not available
Thrombolytic agents (Plasminogen activators)	No recommendation	Cryoprecipitate (initial dose 10 U iv) in thrombolytic agent-associated symptomatic ICH if administered within the previous 24 h. If contraindicated/not available antifibrinolytic agent (tranexamic acid 10–15 mg/kg iv over 20 min or ε-aminocaproic acid 4–5 g iv). If fibrinogen levels < 150 mg/dL, administration of additional cryoprecipitate. Although substitution with fibrinogen concentrate, if available, may be reasonable, there is no recommendation at this time!
Antiplatelet agents	Platelet function testing prior to platelet transfusion is recommended; if laboratory-documented platelet function is within normal ranges or documented platelet resistance. Recommendation against platelet transfusion!	Desmopressin (0.4 μg/kg iv × 1) in ICH with aspirin/cyclooxygenase-1 or adenosine diphosphate (ADP) inhibitors Platelet concentrates in aspirin- or ADP inhibitor-associated ICH in case of neurosurgical intervention. An initial dose of one single donor apheresis unit of platelets. Platelet testing is suggested prior to repeated platelet transfusion and repeated dosing only if persisting abnormalities!

Options and current recommendations for the reversal of antithrombotic agents in adult patients with intracranial hemorrhage (ICH) in the context of TBI. The recommendations are in agreement with the guidance document for novel oral anticoagulants from the International Society of Thrombosis and Hemostasis (ISTH). All antithrombotics (VKAs, Direct factor Xa inhibitors, DTIs, heparins (unfractionated and LMWHs), pentasaccharides, thrombolytics and antiplatelet agents) should be discontinued when ICH is present or suspected. ADP = Adenosine di-phosphate; DIC = Disseminated intravascular coagulation; DTI = Direct thrombin inhibitors; FFP = Fresh frozen plasma; ICH = Intracranial hemorrhage; INR = International Normalized Ratio; LMWH = Low molecular weight heparin; PCC = Prothrombin complex concentrate; rFVIIa = activated recombinant factor FVII; U = unit; VKA = Vitamin K antagonist. First published by Maegele et al. [3] with permission from Elsevier; modified from Maegele et al. [7], with permission from Wolters Kluwer Health, Inc.

## Data Availability

No new data were created. Data analyse has taken place in Institute for Research in Operative Medicine (IFOM), University Witten/Herdecke, Campus Cologne-Merheim, Ostmerheimerstr. 200, D-51109 Köln, Germany.
